# Spatiotemporal Dynamics of Mesozooplankton Trophic Structure and Food Web Configuration in the Vicinity of Daya Bay Nuclear Power Plant

**DOI:** 10.3390/microorganisms14010203

**Published:** 2026-01-15

**Authors:** Yanjiao Lai, Bingqing Liu, Mianrun Chen

**Affiliations:** 1 South China Sea Development Research Institute, Ministry of Natural Resources (Remote Sensing Technology Application Center of South China Sea, MNR), Southern Marine Science and Engineering Guangdong Laboratory (Zhuhai), Guangzhou 510300, China; 2 Key Laboratory of Marine Ecological Monitoring and Restoration Technologies\East China Sea Ecology Center, Ministry of Natural Resources, Shanghai 201206, China; 3 School of Oceanography, Shanghai Jiao Tong University, Shanghai 200030, China

**Keywords:** mesozooplankton, trophic structure, planktonic food web, stable carbon and nitrogen isotopes, Daya Bay nuclear power plant

## Abstract

Mesozooplankton play a pivotal role in marine pelagic food webs, mediating energy and matter transfer between primary producers and higher trophic levels. Daya Bay, a semi-enclosed bay located in the northern South China Sea, has undergone significant environmental changes due to anthropogenic activities, such as thermal discharge from nuclear power plants and eutrophication. This study examined the mesozooplankton community structure, feeding preferences, and food web organization through four seasonal cruises (May 2022, February 2023, August 2023, and November 2023), employing stable isotope analysis and a Bayesian Isotopic Mixing Model. Results indicate that mesozooplankton abundance and diversity were lower in regions affected by thermal discharge, suggesting a suppressive effect of elevated temperatures. Seasonal shifts in dominant species were observed: *Penilia avirostris* and *Dolioletta gegenbauri* dominated the community in spring, while *Noctiluca scintillans* blooms occurred in summer and winter. Isotopic analysis revealed distinct trophic strategies: copepods exhibited omnivorous habits, whereas cladocerans and tunicates showed stronger herbivorous tendencies. *N. scintillans* functioned as a high-trophic omnivore, preying on copepod larvae and competing for food resources. Overall, the mesozooplankton community was characterized by an omnivory-dominated trophic network, which enhanced resilience yet remains sensitive to anthropogenic disturbances. This study clarifies how human-induced environmental changes reshape trophic pathways in subtropical coastal waters, providing a valuable reference for long-term monitoring and ecosystem management in Daya Bay.

## 1. Introduction

Mesozooplankton (200–2000 μm) play a crucial role in pelagic food webs owing to their high abundance and intermediate trophic position linking primary producers and microbial food web to higher trophic levels [1,2]. They transfer matter and energy to upper trophic levels and regulate lower ones through feeding, serving as a key component of the classical marine food chain (phytoplankton–zooplankton–fish) [3,4]. Through top-down control, mesozooplankton directly reduce prey biomass and indirectly influence primary producers via trophic cascades. Therefore, understanding their ecological role is fundamental for evaluating the dynamics and stability of marine food webs.

The ecological significance of mesozooplankton within planktonic food webs is largely determined by their trophic levels relative to primary producers. These trophic levels reflect a balance among groups with different feeding strategies and are ultimately shaped by species composition. Identifying the trophic positions of mesozooplankton, whether herbivorous, omnivorous, or carnivorous, is thus essential for elucidating predator–prey interactions and for understanding the overall organization of planktonic food webs.

Daya Bay is a semi-enclosed bay situated in the northern part of the South China Sea. In recent years, rapid social and economic development has led to significant environmental degradation in the bay. The expansion of fishery activities and fertilizer pollution have caused eutrophication of seawater [5]. In addition, thermal discharge from the nuclear power plant has resulted in extensive warming of local waters [6]. These environmental changes have had exerted profound impacts on the marine ecosystem [7,8,9]. In particular, the mesozooplankton community, which is highly sensitive to environmental variation, has undergone remarkable structural shifts. Dominant species have changed, driven by the rapid rise in water temperature and increased food supply in Daya Bay [10,11]. Large-scale zooplankton blooms have frequently occurred in Daya Bay, associated with variations in temperature, salinity, and food availability [12,13].

Although pronounced shifts in the mesozooplankton community structure of Daya Bay have been well documented, their ecological consequences remain poorly understood. This knowledge gap arises because previous studies have mainly focused on describing community composition while paying insufficient attention to changes in ecological functions. As a result, critical aspects such as mesozooplankton feeding preferences, trophic relationships, and food web structure in Daya Bay remain unclear, hindering a comprehensive understanding of the ecological effects of these community changes. To address this issue, a systematic investigation of mesozooplankton feeding ecology and food web configuration is required. However, the taxonomic complexity, broad size spectrum, and trophic versatility of zooplankton pose challenges to accurately delineating their food web structure.

Stable isotope analysis has become a widely used and reliable tool for investigating zooplankton trophic ecology and food web structures in marine ecosystems [14,15,16]. The carbon isotope ratio (δ^13^C) identifies food sources because of minimal fractionation (0.5–1‰ per trophic level), whereas the nitrogen isotope ratio (δ^15^N) estimates relative trophic levels, as the δ^15^N in tissues of consumers is typically 2–3‰ higher than that in their prey [17,18,19]. Together, these tracers provide insights into prey selectivity, feeding history, and spatial variations in mesozooplankton communities. Advances in isotopic mixing models have further improved the quantitative estimation of source contributions and the reconstruction of energy flow within marine planktonic food webs [20,21]. However, due to the highly complex taxonomic composition of mesozooplankton, isolating each species to determine the corresponding isotope values is impractical, as this requires considerable expertise and an extremely large number of samples. Therefore, Chen et al. [22] used a mass-balancing linear mixing model combined with community structure data to retrodict the isotope values of the entire community, enabling the estimation of isotope values for different species or groups.

This study focused on representative areas of Daya Bay adjacent to the nuclear power plant and systematically examined the mesozooplankton community structure, feeding relationships, and food web configuration using a Bayesian Isotopic Mixing Model. By elucidating the trophic levels, feeding interactions, and dietary preferences of mesozooplankton under anthropogenic stress, this study reveals how human-induced environmental changes reshape the trophic pathways and ecological roles of mesozooplankton. The findings will provide essential baseline parameters for understanding how human activities alter the ecological functions of mesozooplankton in Daya Bay, thereby contributing to the assessment of ecosystem stability and the development of effective strategies for coastal ecosystem management under ongoing environmental stress.

## 2. Materials and Methods

### 2.1. Study Area and Time

**Figure 1:**
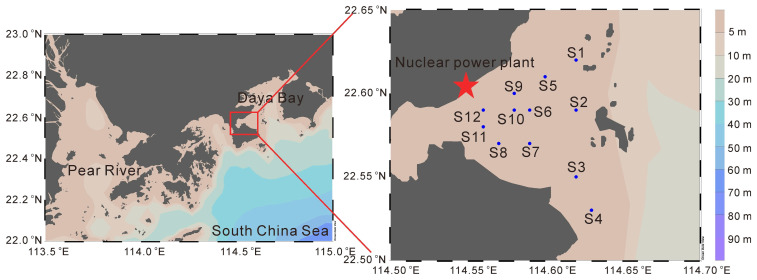
The location of sampling stations in this study (dots in the map indicate sampling stations).

This study took the thermal discharge area of the Daya Bay Nuclear Power Plant and its surrounding sea area as the experimental site (Figure 1). Four cruises were conducted in May 2022 (spring), February 2023 (winter), August 2023 (summer), and November 2023 (autumn).

### 2.2. Field Sampling and Trawling

Environmental Parameter Measurement: A multi-parameter water quality analyzer (EXO2, YSI, Yellow Spring, OH, USA) was used to measure water quality parameters such as water temperature and salinity at 12 stations.

Seawater Collection: A 5 L water sampler was used to collect sufficient water samples from the sea surface (at 0.5 m depth) and place them in a 20 L PC barrel. The water samples were filtered through a 200 μm aperture silk screen to remove mesozooplankton.

Analysis of Basic Chemical Parameters: (1) Nutrients: Parallel 80 mL seawater samples filtered through the silk screen were taken from the 20 L PC barrel and placed in transparent plastic bottles. These samples were first stored in a −20 °C refrigerator and then transferred to a −80 °C refrigerator in the laboratory. Subsequently, the nutrient concentrations, mainly including nitrate, nitrite, ammonia nitrogen, and phosphate, were measured in the laboratory using an intermittent chemical analyzer (SmartChem-200 model, Westco Scientific Instruments, AMS Alliance S.p.A., Guidonia Montecelio, Rome, Italy). Nitrate, nitrite, and ammonia nitrogen together are referred to as Dissolved Inorganic Nitrogen (DIN). (2) Particle Organic Carbon (POC): Parallel 1 L seawater samples filtered through the silk screen were filtered through a GF/C membrane (47 mm in diameter). The GF/C membrane was baked in a muffle furnace at 450 °C for 4 h and weighed before filtration. After filtration, the GF/C membrane containing the sample was wrapped with tin foil and stored in a liquid nitrogen tank. In the laboratory, the sample was stored in a −80 °C refrigerator. Subsequently, the sample was freeze-dried using a freeze-dryer (FDU-1200 model, TOKYO RIKAKIKAI CO., LTD., Tokyo, Japan), and the GF/C membrane with the sample was weighed. The sample was compacted, wrapped with a tin boat, placed in a sample well plate, and the carbon and nitrogen contents in the sample were determined using an elemental analyzer (Vario isotope cube, Elementar Analysensysteme GmbH, Langenselbold, Hesse, Germany).

Biological Parameters: Chlorophyll *a*: 0.5~1 L of filtered seawater was passed through a 20-μm-aperture nylon membrane, a 2-μm-aperture polycarbonate membrane, and a 0.7-μm-aperture GF/F glass-fiber membrane successively, and then wrapped with tin foil and stored in a liquid nitrogen tank. In this paper, phytoplankton with a size range of 20~200 μm is defined as small-sized phytoplankton (hereinafter referred to as microplankton), 2~20 μm as nano-sized phytoplankton (hereinafter referred to as nanoplankton), and 0~2 μm as pico-sized phytoplankton (hereinafter referred to as picoplankton). In the laboratory, the membrane was placed in a 15 mL centrifuge tube containing 5 mL of 90% acetone and stored in a 4 °C refrigerator in the dark for 24 h to extract the chlorophyll *a* from the membrane. The fluorescence value of the sample was measured using a fluorometer (Triology Model 7200, Turner Designs, San Jose, CA, USA). The chlorophyll *a* concentration of size-fractionated phytoplankton was calculated according to the parameters obtained from the fluorescence-chlorophyll standard curve (microChl *a*: chlorophyll *a* concentration of microplankton; nanoChl *a*: chlorophyll *a* concentration of nanoplankton; picoChl *a*: chlorophyll *a* concentration of picoplankton; totalChl *a*: the total chlorophyll *a* concentration).

Identification and Counting of mesozooplankton Species: A customized plankton net (200 μm aperture, 0.5 m inner diameter) was used for vertical trawling (from 0.5 m above the bottom to the surface). A flow meter was tied to the net mouth to record the flow meter reading. The mesozooplankton collected by trawling was fixed in a 500 mL plastic bottle with 5% formalin. In the laboratory, a stereomicroscope (SMZ-25, Nikon, Tokyo, Japan) and an optical microscope (DM2000 LED, Leica, Wetzlar, Germany) were used to count and identify the mesozooplankton species. Species identification was conducted with reference to standard taxonomic keys and authoritative guides [23,24].

Dry Weight Biomass of Mesozooplankton: Vertical trawling was also carried out using a plankton net, and the flow meter reading was recorded. A part of the water sample containing mesozooplankton was filtered through a pre-weighed 10 μm-aperture PC membrane. The PC membrane was placed in a Petri dish, covered, and the volume of the water sample was recorded. In the laboratory, the PC membrane was dried in an oven at 60 °C for four hours and then weighed to determine the dry weight of mesozooplankton. The subsequent methods for determining the stable carbon and nitrogen isotope ratios were the same as those for the seawater samples filtered through the silk screen.

Carbon and Nitrogen Stable Isotopes: (1) Water Samples, 1 L of filtered seawater was filtered through a 47 mm-diameter, 1.2 μm-aperture GF/C membrane that had been baked in a muffle furnace at 450 °C for 4 h and weighed. After filtration, the GF/C membrane containing the sample was wrapped with tin foil and stored in a liquid nitrogen tank. In the laboratory, the sample was stored in a −80 °C refrigerator. (2) Meszooplankton Samples: Zooplankton samples were obtained by trawling using the above-mentioned method, filtered through a fired and weighed GF/C membrane, wrapped with tin foil, stored in a liquid nitrogen tank, and the volume of the corresponding water sample was recorded. (3) Analysis: The filter membranes containing water or zooplankton samples were sent to Guangzhou Anengte Chemical Technology Co., Ltd. to determine the stable carbon and nitrogen isotope ratios in the samples. The filter membrane sample was placed in a disposable ceramic crucible with micropores. It was soaked in 1 N HCl for 1 h to remove inorganic carbon, and then eluted 3–5 times with ultrapure water until the pH value of the leachate was neutral. After the water was filtered dry, the acid-treated filter membrane sample was taken out of the disposable ceramic crucible and placed in a vacuum freeze-dryer for 48 h for freeze-drying. The dried filter membrane sample was unfolded, and the inner layer of the filter membrane with the sample attached was carefully peeled off with tweezers. The inner layer of the filter membrane was wrapped with a tin boat and placed in an automatic sampling tray for testing. The stable carbon and nitrogen isotope ratios of the samples were determined using a method that combines an elemental analyzer (Vario Isotope Cube, Elementar Analysensysteme GmbH, Langenselbold, Hesse, Germany) and a stable isotope ratio mass spectrometer (model IsoPrime 100, Elementar UK Ltd., Stockport, Greater Manchester, UK).

### 2.3. Data Analysis

This study referred to the method of Chen et al. [22] to estimate the stable carbon and nitrogen isotopes of classified mesozooplankton and size-fractionated plankton in the plankton community at each experimental station. Taking mesozooplankton as an example, it was assumed that the proportion selected for isotope analysis was the same as the proportion used for community structure analysis in the sample [22]. The stable carbon and nitrogen isotope ratios of each classified mesozooplankton were calculated according to multiple linear regression. The main calculation methods are as follows:

(1) $$m\;\times \;\delta^{13}C\;={\;m}_{1}\;\times \;\delta^{13}C_{1}\;+\;m_{2}\;\times \;\delta^{13}C_{2}\;+\;m_{3}\;\times \;\delta^{13}C_{3}\;+\;\ldots \;+\;m_{x}\;\times \;\delta^{13}C_{x}\;+\;error$$

(2) $$m\;\times {\;\delta }^{15}N=m_{1}\;\times \;\delta^{15}N_{1}+m_{2}\;\times {\;\delta }^{15}N_{2}+{\;m}_{3}\;\times \;\delta^{15}N_{3}+\ldots +m_{x}\;\times {\;\delta }^{15}N_{x}+error$$

In Formulas (1) and (2), m represents the total biomass of the mesozooplankton community, and $m_{1}$~$m_{x}$ represent the biomasses of various groups/populations. $\delta^{13}C$ and $\delta^{15}N$ are the stable carbon and nitrogen isotope ratios of the mesozooplankton community obtained by trawling at the experimental stations, as measured by the above-mentioned instruments. $\delta^{13}C_{1}$~$\delta^{13}C_{x}$ and $\delta^{15}N_{1}$~$\delta^{15}N_{x}$ are the stable carbon and nitrogen isotope ratios of each mesozooplankton group/population, respectively. “error” represents the error of the linear regression. The above calculations are completed through the linear regression equation (lm function) in R software (R version 4.3.3, R Core Team, Vienna, Austria). By inputting the known data columns of $\delta^{13}C$ and $\delta^{15}N$ (12 stations in each season), as well as m and $m_{1}$~$m_{x}$, the stable carbon isotope ratios $\delta^{13}C_{1}$~$\delta^{13}C_{x}$, the average stable nitrogen isotope ratios $\delta^{15}N_{1}$~$\delta^{15}N_{x}$ and the standard errors of each mesozooplankton group/population can be estimated.

In this study, the total biomass of the mesozooplankton community (m) is characterized by dry weight (measured as described above), and the biomasses of various groups/populations are realized by their proportion of the total dry weight $m_{1}$~$m_{x}$. Since it is difficult to obtain the dry weights of each group and population, we estimate them by combining the abundance and the proportion of individual theoretical biomass. First, we list the body-length ranges of the observed species, and then obtain their individual theoretical biomass based on established empirical relationships between body length and biomass (dry weight) for each species [25,26,27]. Second, based on the abundance of each identified species multiplied by the individual theoretical biomass of each group/population, we obtain the theoretical biomass of each group/population and the proportion of each population in the total mass. According to the actual dry-weight value obtained by trawling at the experimental stations, the dry weights of each group/population are obtained in proportion. For the convenience of statistics, according to the frequency of group occurrence, the proportion of biomass and the differences in species (that is, similar species with low biomass or low occurrence frequency are combined into one category, and species with relatively high biomass proportion are classified separately), the mesozooplankton community is divided into Dinoflagellata (in this study, specifically referring to *Noctiluca scintillans*), Copepoda, Cladocera, other Crustaceana (including Ostracoda, Decapoda, Cirripedia, Mysida, etc.), Tunicates, Larvae (mainly including Echinodermata, Echinopluteus larva, Auricularia larva, Bipinnaria larva, Cnidaria, Tubularia larva, Phoronida, Actinotrocha larva, Sabellaria larva and fish eggs) and Other Zooplankton. Other Zooplankton includes Cnidaria, Annelida, Mollusca and Chaetognatha.

Regarding the δ^13^C and δ^15^N of size-fractionated plankton, since the size-fractionated plankton in the water samples was not collected by hierarchical filtration in this experiment, the biomass of size-fractionated phytoplankton is used as the biomass of size-fractionated plankton (microplankton, nanoplankton, and picoplankton). The calculation is also carried out using Formulas (1) and (2). The measured chlorophyll-*a* concentrations of size-fractionated plankton are used as $m_{1}$~$m_{x}$ (in this study, it is divided into 3 levels, that is x = 3), the total chlorophyll-*a* concentration of plankton is m. The average values and standard errors of the δ^13^C and δ^15^N of size-fractionated plankton are estimated in R software(R version 4.3.3, R Core Team, Vienna, Austria) using the above-mentioned method.

After obtaining δ^15^N of each mesozooplankton category/population and size-fractionated phytoplankton, their trophic levels can be determined, respectively, through the following formulas:

(3) $$TL\;=\;1\;+\;(\delta^{15}N-\delta^{15}N_{basal})/3$$

In Formula (3), *TL* represents the trophic level of the consumer, $\delta^{15}N$ is the stable nitrogen isotope ratio of various groups and species, and $\delta^{15}N_{basal}$ is the stable nitrogen isotope ratio of the basal trophic level. In this project, picoplankton is taken as the basal trophic level. Since the $\delta^{15}N$ of various groups/species obtained through Formulas (1) and (2) only have the mean value and the standard deviation (*sd*), when calculating the standard deviation (sdTL), we sum up the standard error of the δ^15^N of the target population/group (${sd}_{1}$) and the standard error of the δ^15^N of the basal trophic level (${sd}_{2}$) (${sd}_{TL}\;=\;\sqrt{{{sd}_{1}}^{2}+{{sd}_{2}}^{2}}/3$).

To analyze the omnivory of various groups/populations, this study uses the Bayesian isotopic mixing model to estimate the relative contributions of phytoplankton of different particle sizes and lower trophic levels to the diet of higher-trophic-level consumers [21,22,28]. The model assumes that the stable isotope ratio of each consumer follows a Gaussian distribution with unknown mean and standard deviation. The mean value of the consumer’s stable isotope is obtained by the weighted combination of the stable isotope ratios of different food sources (Sources) and the fractionation factor. The weights of various food sources are the proportions of food sources in the diet. The model formula is as follows:

(4) $$\delta^{13}C_{consumer}\;=\;f_{1}\;\times \;\delta^{13}C_{source\;1}\;+\;f_{2}\;\times \;\delta^{13}C_{source\;2}\;+\;f_{3}\;\times \;\delta^{13}C_{source\;3}\;+\;\cdots \;+{\;f}_{n}\;\times \;\delta^{13}C_{source\;n}\;+\;\alpha_{carbon}$$

(5) $$\delta^{15}N_{consumer}={\;f}_{1}\;\times \;\delta^{15}N_{source\;1}+f_{2}\;\times {\;\delta }^{15}N_{source\;2}+f_{3}\;\times {\;\delta }^{15}N_{source\;3}+\cdots f_{n}\;\times {\;\delta }^{15}N_{source\;n}+\alpha_{nitrogen}$$

(6) $$f_{1}+{\;f}_{2}+{\;f}_{3}+\cdots +f_{n}=1$$

In the formula, $\delta^{13}C_{consumer}$ and $\delta^{15}N_{consumer}$ are the stable carbon and nitrogen isotope ratios of the consumer, respectively. $\delta^{13}C_{source\;1}$*~*$\delta^{13}C_{source\;n}$ and $\delta^{15}N_{source\;1}$*~*$\delta^{15}N_{source\;n}$ are the stable carbon and nitrogen isotope ratios of each food source, respectively. $f_{1}$~$f_{n}$ are the proportions of different food sources. $\alpha_{carbon}$ is the sum of the fractionation factors, also known as trophic enrichment factors, of the stable carbon isotope ratios of all food sources. $\alpha_{nitrogen}$ is the sum of the trophic enrichment factors of the stable nitrogen isotope ratios of all food sources. The calculation formulas for $\alpha_{carbon}$ and $\alpha_{nitrogen}$ are as follows:

(7) $$\alpha_{carbon\;}=\;\sum_{i=1}^{n}(TL_{consumer}-L_{source\;i})\;\times \;0.5‰$$

(8) $$\alpha_{nitrogen}=\sum_{i=1}^{n}(TL_{consumer}-TL_{source\;i})\;\times \;3‰$$

This model is implemented using the open-source R package “Stable isotope analysis package” (SIAR, version 4.2) [21] in R version 4.3.3 (R Core Team, Vienna, Austria). In this study, the stable isotope ratios of consumers obtained from the multiple linear regression model are expressed as means and standard errors. Therefore, a vector consisting of 250 numbers was generated for each group of consumers through a random normal distribution function. Then, we need to organize the isotope ratios of different food sources for different consumers [results calculated by formulas (1) and (2)] and the fractionation factors for different food sources (the mean values and standard errors of $\alpha_{carbon}$ and $\alpha_{nitrogen}$, and the calculation method of the standard error is ${sd}_{\alpha }\;=\;\sqrt{{{sd}_{TL1}}^{2}+{{sd}_{TL2}}^{2}}$). The model runs mainly based on the default number of iterations provided by the SIAR package (iterations = 500,000, burnin = 50,000). Model fitting is accomplished through the Markov Chain Monte Carlo (MCMC) method. This method is used to simulate reasonable values for the proportion of each food source in the diet [21,28].

In this study, the calculations of the stable isotope ratios of mesozooplankton groups/populations and size-fractionated phytoplankton, as well as the estimation of the food sources of plankton community consumers, are all completed using R software version 4.0.4. One-way analysis of variance or independent *t*-test in SigmaPlot 15.0 is used for difference analysis, with a confidence interval of 95%. When *p* < 0.05, the difference is statistically significant; when *p* < 0.01, the difference is highly significant. For non-normally distributed samples, Kruskal–Wallis one-way analysis of variance or Mann–Whitney test is used for difference analysis, with a confidence interval of 95%.

Redundancy analysis (RDA) was performed to investigate relationships between the mesozooplankton abundance and environmental variables using the R package “vegan” (version 2.0-3) [29] in R version 4.3.3 (R Core Team, Vienna, Austria). Prior to RDA, the abundances of the mesoplankton groups were transformed using Hellinger transformations, and the environmental variables data were normalized. Hierarchical partitioning analysis based on the RDA were performed to evaluate the independent contributions of each environmental variables on the distribution of mesoplankton groups, using the R package “rdacca.hp” (version 1.1.6) [30] in R version 4.3.3 (R Core Team, Vienna, Austria). Correlation analysis was conducted to examine relationships between the environmental variables, the total mesozooplankton abundance, and the Shannon–Wiener index.

## 3. Results

### 3.1. Environmental Parameters

Environmental factors exhibited pronounced seasonal variations. Water temperature was significantly higher in summer (29.75 ± 0.58 °C; *p* < 0.0001) and lowest in winter (18.98 ± 1.04 °C; *p* < 0.0001). Salinity showed the opposite trend, with significantly higher values in winter (33.74 ± 0.23; *p* < 0.0001) and the lowest in spring (31.69 ± 0.89; *p* < 0.01). Nitrate concentrations were significantly elevated in summer (0.02 ± 0.00 mg L^−1^) and winter (0.02 ± 0.00 mg L^−1^) compared with the other seasons (*p* < 0.001). Similarly, ammonia concentrations reached their maximum in winter (0.09 ± 0.05 mg L^−1^; *p* < 0.05). Phosphate concentrations were significantly higher in autumn (0.18 ± 0.22 mg L^−1^; *p* < 0.01), remaining relatively low during the other seasons.

**Figure 2:**
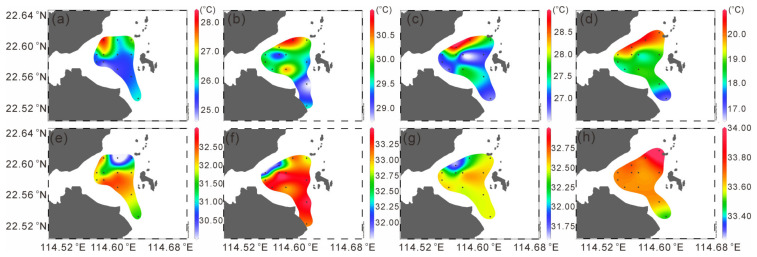
Distribution of the sea surface temperature (**a**–**d**) and sea surface temperature salinity (**e**–**h**) (dots in the map indicate sampling stations).

In each season, the surface temperatures in the area near the nuclear power plant were higher than that in other areas, suggesting the impact of thermal discharge. In spring, the surface temperature near the nuclear power plant was relatively high, reaching 28.15 °C, while the lowest temperature was at station S8 on the south side, which was 24.94 °C (Figure 2a). In summer, the overall temperature in the study area was relatively high. The highest temperature was at station S7, which was 30.45 °C. Station S9 near the nuclear power plant had a temperature of 30.27 °C, and the lowest temperature was at station S3 far from the nuclear power plant, which was 28.76 °C (Figure 2b). In autumn, the highest temperature was at station S9, which was 28.93 °C, and the lowest was at station S6, which was only 26.51 °C (Figure 2c). In winter, the highest temperature was at station S5, which was 20.53 °C, and the lowest was at station S4, which was 16.97 °C (Figure 2d).

Overall, the spatial distribution trend of salinity is opposite to that of temperature. Except in winter, the salinity on the southwest side of the study area is the highest, while that at stations near the nuclear power plant is relatively low (Figure 2e–g).

**Figure 3:**
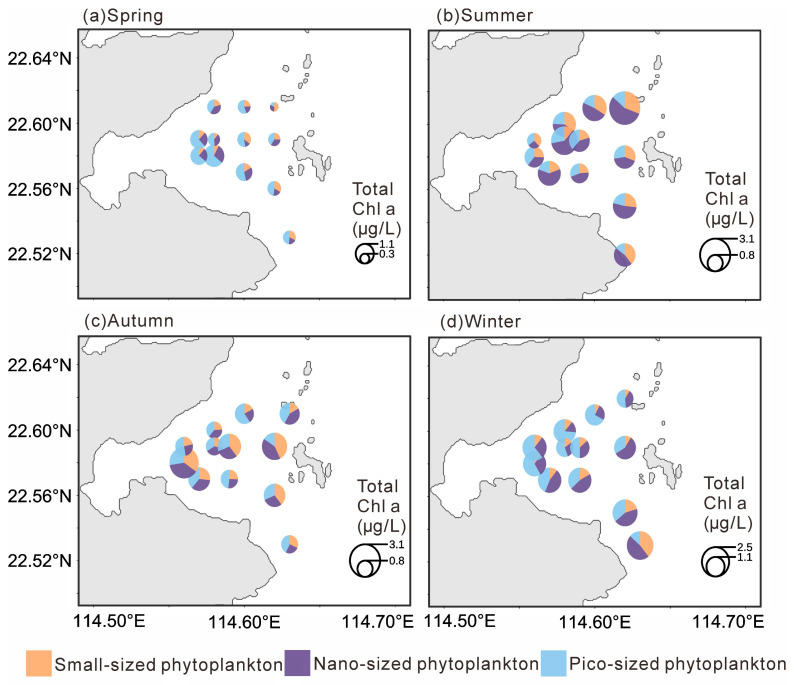
Distribution of the size-fractionated chlorophyll *a* concentration in four seasons.

Chlorophyll *a* concentrations also displayed distinct seasonal variations (Figure 3). Total Chlorophyll *a* was significantly lower in spring (0.72 ± 0.32 µg L^−1^; *p* < 0.001), with no significant differences among the other seasons. MicroChl *a* were significantly higher in summer (0.48 ± 0.23 µg L^−1^) and autumn (0.45 ± 0.29 µg L^−1^; *p* < 0.01) than in other seasons. NanoChl *a* was significantly higher in summer (0.85 ± 0.45 µg L^−1^) and winter (0.63 ± 0.30 µg L^−1^; *p* < 0.05). PicoChl *a* reached its highest level in winter (0.77 ± 0.29 µg L^−1^; *p* < 0.05), while differences among the other seasons were not significant.

In spring, totalChl *a* showed a decreasing trend from west to east, with the highest value observed in the southwestern area (1.44 µg L^−1^). PicoChl *a* dominated in the western stations, whereas microChl *a* was more abundant in the eastern part. In summer, totalChl *a* were generally high with little spatial variation, reaching a maximum in the northeastern region (3.30 µg L^−1^), and were mainly dominated by nanoChl *a*. During autumn, totalChl *a* was relatively higher in the southwestern area, with a maximum of 2.88 µg L^−1^, but was generally lower near the nuclear power plant; microChl *a* was the dominant size fraction during this season. In winter, total Chl *a* remained low in the vicinity of the nuclear power plant (as low as 0.96 µg L^−1^) but gradually increased southward, reaching up to 2.39 µg L^−1^. PicoChl *a* dominated in the western region, while nanoChl *a* was dominant in the eastern region.

### 3.2. Mesozooplankton Community

#### 3.2.1. Abundance and Diversity of Mesozooplankton

**Figure 4:**
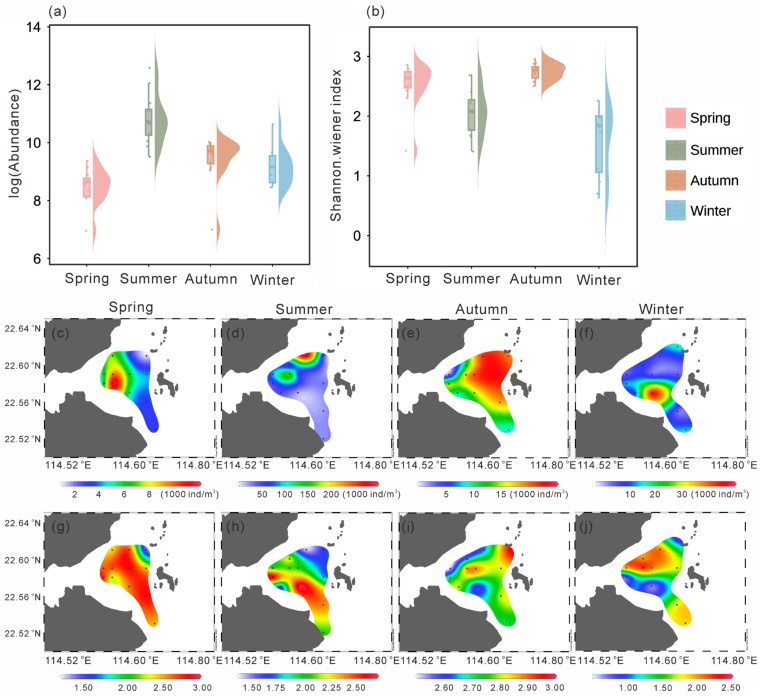
Abundance (**a**) and Shannon–Wiener index (**b**) values in four seasons. Spatial distribution of abundance (**c**–**f**) and Shannon–Wiener index (**g**–**j**) in four seasons (dots in the map indicate sampling stations).

Mesozooplankton abundance exhibited pronounced seasonal variations (Figure 4a). The mean abundance in spring was the lowest among the four seasons (5605.32 ± 2927.39 ind. m^−3^; *p* < 0.05), whereas the highest mean abundance occurred in summer (73,423.79 ± 80,982.45 ind. m^−3^; *p* < 0.01). Spatially, higher abundances in spring were observed in the southwestern area, while lower values occurred in the northern high-temperature zone and the southeastern region, with the lowest abundance (1048.55 ind. m^−3^) recorded in the northeast (Figure 4c). In summer, zooplankton abundance increased markedly across the study area, reaching a maximum (292,447.18 ind. m^−3^) in the northwest (Figure 4d). During autumn, low-abundance zones appeared near the nuclear power plant in the northwest (minimum: 1094.54 ind. m^−3^), whereas higher values were found in the eastern region (Figure 4e). In winter, abundance was generally low in the north (minimum: 4715.09 ind. m^−3^) and relatively high in the southwest, with a maximum of 15,714.45 ind. m^−3^ (Figure 4f).

The Shannon–Wiener index also showed clear seasonal variation (Figure 4b), with significantly lower values in summer and winter compared with the other two seasons (*p* < 0.01). In spring, the Shannon–Wiener index generally exceeded 2.50, except for a minimum value of 1.42 in the northeast (Figure 4g), coinciding with areas of higher temperature (Figure 2a) and lower abundance (Figure 4a). Similarly, in summer, the Shannon–Wiener index was lowest in the northeast (1.41) and increased gradually toward the south (Figure 4h). In autumn, the Shannon–Wiener index was generally higher across the study area but showed a local minimum (2.50) in the northwest (Figure 4i), again corresponding to a high-temperature zone (Figure 2c), and reached a maximum of 2.96 in the southeast. In winter, the Shannon–Wiener index was generally low, particularly in the northern and southwestern areas, where it reached a minimum of 0.63, while slightly higher values occurred in the central and southeastern regions (Figure 4j).

#### 3.2.2. Species Composition of Mesozooplankton

**Figure 5:**
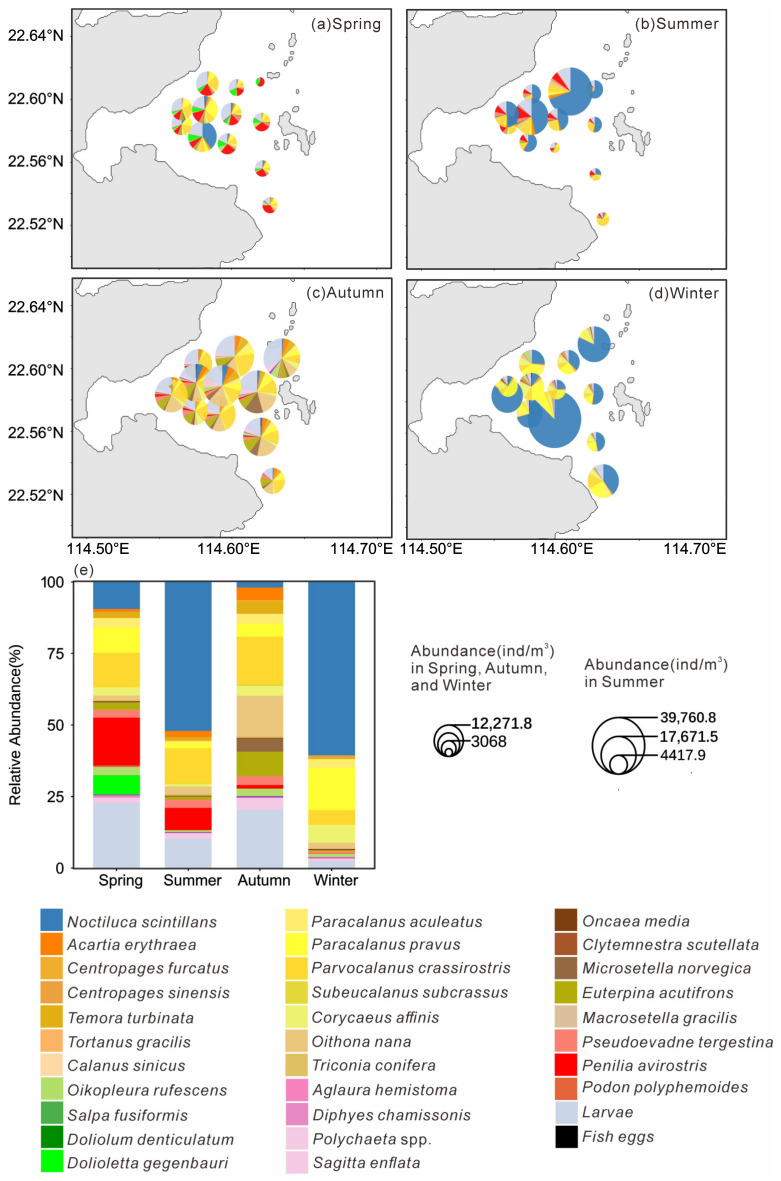
Spatial distribution of each mesoplankton species (**a**–**d**) and relative abundance of each species in all species (**e**) in four seasons.

The distribution pattern of mesozooplankton across stations in four seasons is presented in Figure 5a–d. In spring, Cladocera accounted for the highest proportion of abundance among all samples (19.91%), reaching the peak level across the four seasons, primarily represented by *Penilia avirostris* (16.71%; Figure 5e). The abundance proportion of *Dolioletta gegenbauri* (6.71%) increased significantly compared to other seasons. In summer, *N. scintillans* had the highest abundance proportion, reaching 52.09%, followed by *Parvocalanus crassirostris* 12.29% and *P. avirostris* 7.73% (Figure 5e). In Autumn, *P. crassirostris* had the highest abundance proportion (16.59%), and the cyclopoid copepod *Oithona nana* ranking second (14.08%), reaching its peak abundance proportion across all four seasons (Figure 5e). In winter, *N. scintillans* accounted for 60.64% of the total abundance, followed by *Paracalanus pravus* (14.67%; Figure 5e).

#### 3.2.3. Relationships Between Mesozooplankton Groups and Environmental Factors

**Figure 6:**
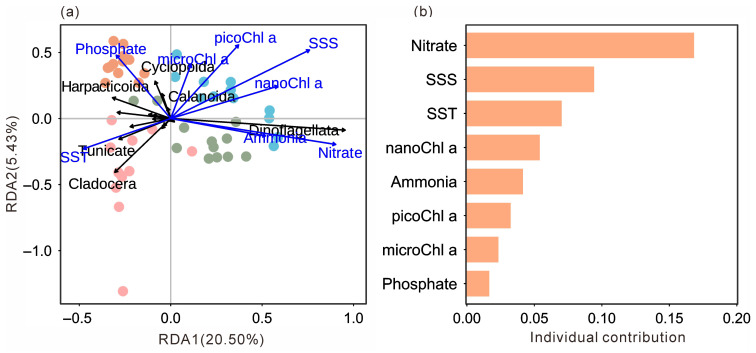
Ordination plot of the redundancy analysis (RDA) based on the abundance of each group and normalized environmental variables (**a**). Hierarchical partitioning analysis based on the RDA results (**b**). SST, sea surface temperature; SSS, sea surface salinity; MicroChl *a*, chlorophyll *a* concentration of small-sized plankton (20~200 μm); NanoChl *a*, chlorophyll *a* concentration of nano-sized plankton (2~20 μm); PicoChl *a*, chlorophyll *a* concentration of pico-sized plankton (0~2 μm). Dots indicate sampling stations and their colors indicate seasons (pink: spring; green: summer; orange: autumn; blue: winter).

Redundancy analysis (RDA) clearly revealed significant associations between mesozooplankton community structure, seasons, and key environmental variables (Figure 6a). The sampling stations from the four seasons were distinctly separated in the ordination space, indicating pronounced seasonal succession in the mesozooplankton community structure. Within this framework, specific correlation patterns were evident. Spring stations were positively associated with SST but negatively correlated with SSS, totalChl a, Nitrate, and Ammonia, and were closely grouped with Cladocera and Thaliacea. In contrast, summer and winter stations showed positive correlations with SSS, chlorophyll a, Nitrate, and Ammonia, but negative correlations with SST, and were tightly clustered with Dinoflagellata. Autumn stations were located in proximity to Phosphate and were closely associated with the copepod orders Calanoida and Cyclopoida. This distribution pattern was consistent with the seasonal dominance of major mesozooplankton group. Hierarchical partitioning analysis further identified Nitrate as the most influential environmental factor shaping this distribution pattern, followed by SSS and SST (Figure 6b).

**Figure 7:**
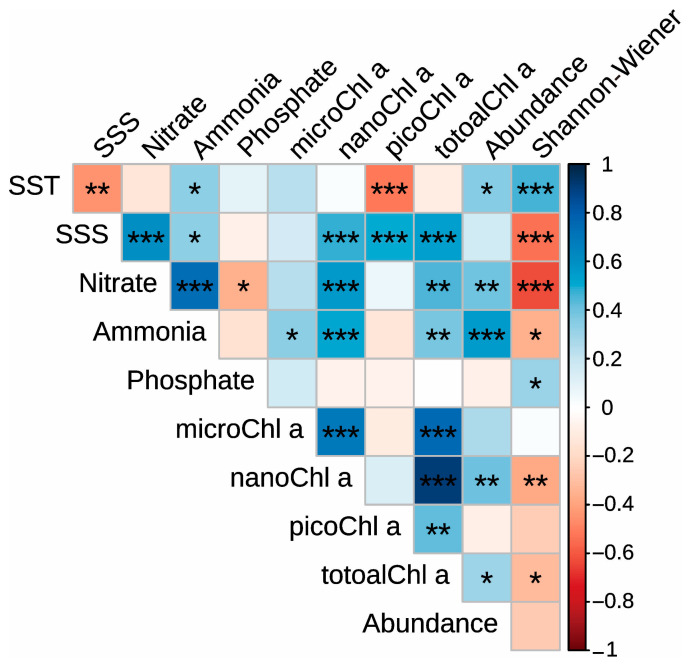
Spearman correlations between the environmental variables, the total mesozooplankton abundance, and the Shannon–Wiener index. “*”, “**” and “***” indicate significance level at < 0.05, *p* < 0.01 and *p* < 0.001, respectively.

Correlation analysis showed that the total mesozooplankton abundance was significantly negatively correlated with SST (*p* < 0.05), Nitrate (*p* < 0.01), Ammonia (*p* < 0.001), nanoChl a (*p* < 0.01), and totalChl a (*p* < 0.05; Figure 7). The Shannon–Wiener index was significantly negatively correlated with SST (*p* < 0.001) and Phosphate (*p* < 0.05) but positively correlated with SSS (*p* < 0.001), Nitrate (*p* < 0.001), Ammonia (*p* < 0.05), nanoChl a (*p* < 0.01), and totalChl a (*p* < 0.05; Figure 7).

### 3.3. Trophic Level Classification of Plankton

#### 3.3.1. Stable Carbon and Nitrogen Isotope Ratios of Size-Fractionated Phytoplankton and Main Mesozooplankton

**Figure 8:**
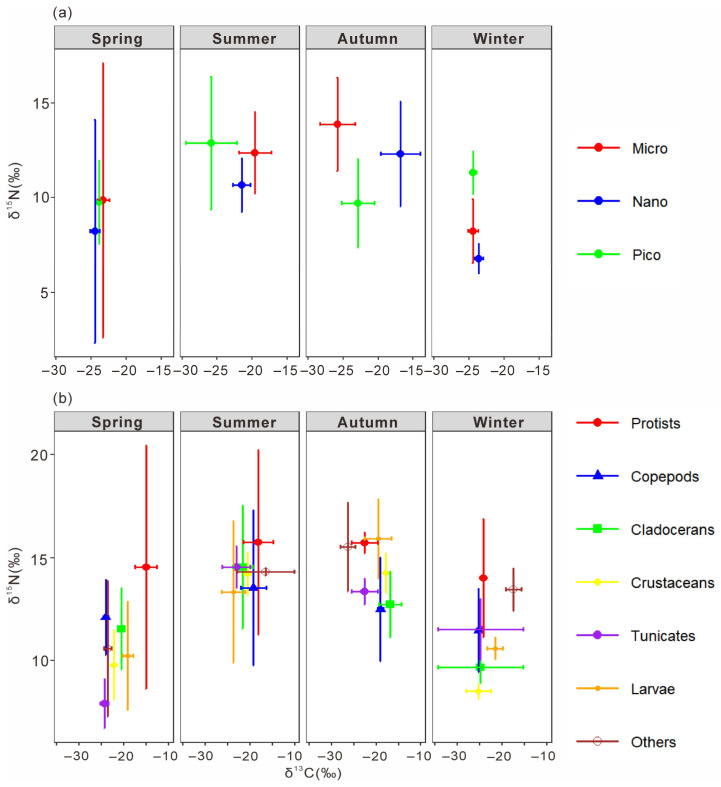
Bi-plots of stable carbon and nitrogen isotope ratios of size-fractionated phytoplankton (**a**) and major mesozooplankton groups (**b**) in the thermal discharge area of Daya Bay Nuclear Power Station in four seasons. Micro, small-sized plankton (20~200 μm); Nano, nano-sized plankton (2~20 μm); Pico, pico-sized plankton (0~2 μm).

The δ^13^C and δ^15^N of size-fractionated phytoplankton and major mesozooplankton groups were estimated using multiple linear regression (Figure 8). The regression models for δ^13^C (R^2^ = 0.9937, *p* < 0.01) and δ^15^N (R^2^ = 0.9208, *p* < 0.01) were both significant, with intercepts of −0.74 ± 0.49‰ and −1.34 ± 0.91‰, respectively.

Significant seasonal variations in δ^13^C and δ^15^N were observed among size-fractionated phytoplankton (one-way ANOVA, *p* < 0.01). In spring, small-sized phytoplankton exhibited significantly higher δ^13^C (−23.31 ± 0.85‰) than nano- and pico-sized fractions, while δ^15^N ranged from 8.22 ± 5.90‰ to 9.87 ± 7.25‰. In summer, δ^13^C decreased with decreasing cell size, from −19.59 ± 2.29‰ in small-sized to −25.88 ± 3.35‰ in pico-sized phytoplankton, with δ^15^N ranging from 10.66 ± 1.42‰ to 12.89 ± 3.50‰. In autumn, δ^13^C varied greatly among size classes, being highest in nano-sized (−16.81 ± 2.82‰) and lowest in small-sized phytoplankton (−25.82 ± 2.51‰), whereas δ^15^N was highest in small-sized (13.88 ± 2.47‰) and lowest in pico-sized (9.70 ± 2.34‰) fractions. In winter, δ^13^C values were relatively uniform across size classes (−23.6‰ to −24.5‰), while δ^15^N was highest in pico-sized phytoplankton (11.32 ± 1.13‰). Overall, both δ^13^C and δ^15^N exhibited pronounced seasonal and size-dependent differences, indicating distinct isotope fractionation patterns among phytoplankton size classes.

The δ^13^C and δ^15^N of the main mesozooplankton groups were well fitted by multiple linear regression (R^2^ = 0.9962 for δ^13^C, R^2^ = 0.9927 for δ^15^N, both *p* < 0.01; Figure 8b). Distinct seasonal and taxonomic differences were observed (one-way ANOVA, *p* < 0.01). In spring, Dinoflagellata showed the highest δ^13^C (−15.08 ± 0.40‰) and δ^15^N (14.53 ± 5.93‰), significantly higher than other groups, while copepods and cladocerans exhibited intermediate values. In summer, δ^13^C ranged from −23.73 ± 2.64‰ in planktonic larvae to −16.53 ± 6.37‰ in other zooplankton, with no significant δ^15^N difference among taxa (13.3–15.8‰). In autumn, δ^13^C varied widely (−26.39 to −16.92‰), being highest in Dinoflagellata and lowest in other zooplankton, while δ^15^N peaked in planktonic larvae and Dinoflagellata (both >15‰). In winter, δ^13^C was lowest in copepods and other crustaceans (≈−25‰) and highest in other zooplankton (−17.49 ± 1.69‰), whereas δ^15^N was again elevated in Dinoflagellata and other zooplankton (>13‰). Overall, Dinoflagellata consistently showed enriched δ^13^C and δ^15^N values, suggesting a higher trophic position. Copepods, cladocerans, and other crustaceans occupied intermediate levels, while tunicates and planktonic larvae displayed more variable isotopic compositions.

#### 3.3.2. The Trophic Levels and Feeding Habits of Plankton

**Figure 9:**
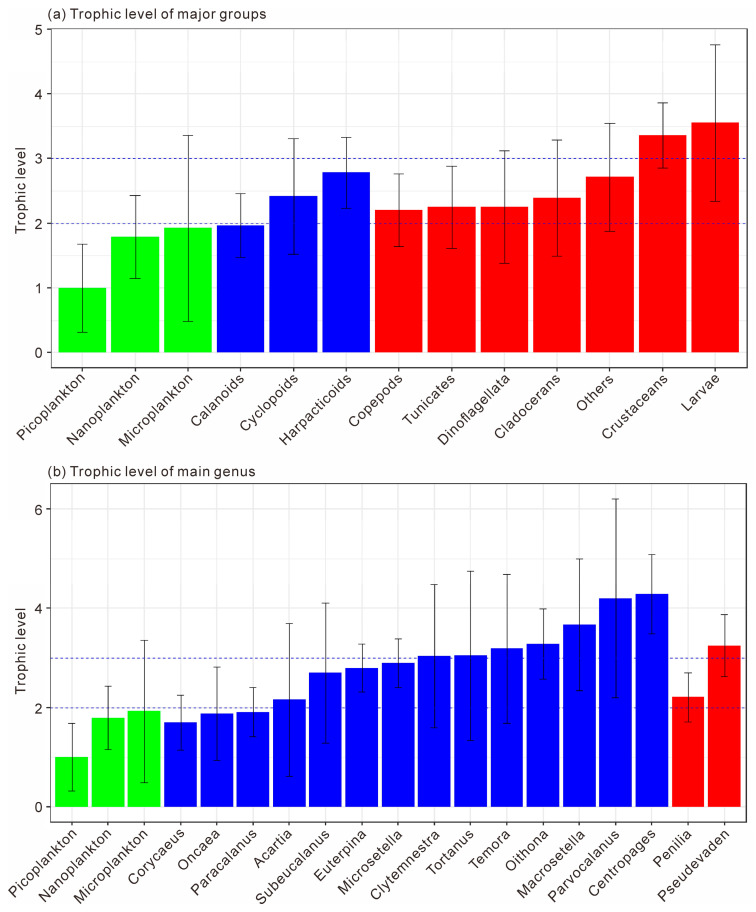
The trophic levels of major planktonic groups (**a**) and major planktonic genus (**b**). Dashed lines of trophic levels at 2 and 3 indicate herbivory and carnivory, respectively. Green bars indicate size-fractionated phytoplankton, blue bars indicate copepods, while red bars indicate cladocerans.

The trophic level classification was based on picoplankton (trophic level = 1). Organisms with trophic levels of 1–2 were considered phytoplankton feeders, 2–3 omnivores, and >3 carnivores. As shown in Figure 9a, microplankton and nanoplankton had trophic levels of 1.79 ± 0.64 and 1.93 ± 1.44, respectively, indicating phytoplankton-feeding behavior. Among zooplankton, Calanoida (1.97 ± 0.49) were also herbivorous, while Cyclopoida (2.42 ± 0.90) and Harpacticoida (2.78 ± 0.55) were omnivorous (F = 4.502, *p* < 0.05). Copepods as a whole had a mean trophic level of 2.20 ± 0.56, reflecting an omnivorous diet with a phytoplankton-feeding tendency. Tunicates, Dinoflagellata, and Cladocerans showed similar omnivorous habits (2.25–2.40), whereas other zooplankton (Cnidaria, Annelida, Mollusca, Chaetognatha) had higher trophic levels (2.72 ± 0.83), indicating carnivory. Other crustaceans (3.36 ± 0.51) and planktonic larvae (3.55 ± 1.21) exhibited the highest values and were significantly more carnivorous than copepods, tunicates, and cladocerans (F = 5.622, *p* < 0.01).

At the genus level (Figure 9b), Corycaeus, Oncaea, and Paracalanus had trophic levels below 2, confirming herbivory (F = 0.323, *p* > 0.05). Acartia, Subeucalanus, Euterpina, and Microsetella ranged from 2.16 to 2.90, representing omnivores, with Microsetella showing a tendency toward carnivory. Clytemnestra, Tortanus, Temora, Oithona, and Macrosetella had trophic levels above 3, suggesting carnivorous feeding, while Parvocalanus and Centropages spp. exceeded 4, representing top carnivores among mesozooplankton. Within Cladocerans, Penilia (2.21 ± 0.50) was omnivorous, whereas Pseudevadne (3.25 ± 0.63) showed carnivorous behavior. Overall, the trophic structure of mesozooplankton in Daya Bay ranged from herbivorous to top carnivorous taxa, reflecting a complex food-web hierarchy.

### 3.4. Food Composition and Feeding Preferences of Mesozooplankton

#### 3.4.1. Food Composition and Feeding Preferences of Mesozooplankton Groups

**Figure 10:**
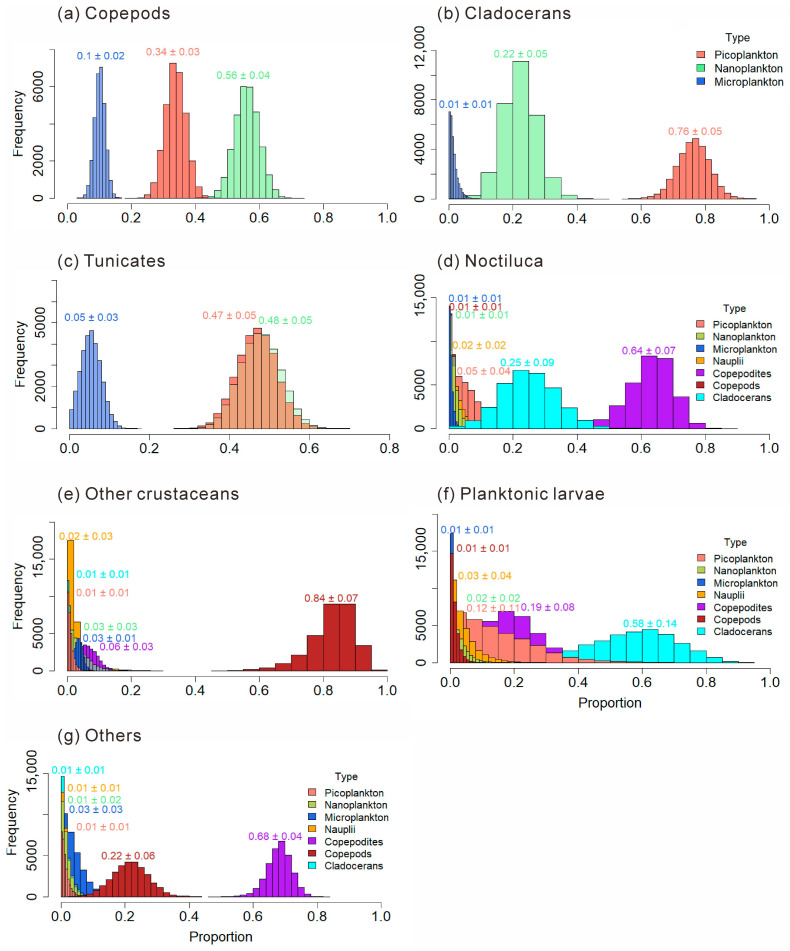
Food composition of main mesozooplankton groups: Copepods (**a**); Cladocerans (**b**); Tunicates (**c**); *Noctiluca scintillans* (**d**); Other crustaceans (**e**); Planktonic larvae (**f**); Other mesozooplankton (**g**).

The food composition proportions of main mesozooplankton groups are shown in Figure 10. Copepods, Cladocerans, and Tunicates have selective feeding preferences for size-fractionated plankton. The omnivorous and phytoplankton-feeding-biased Copepods obtained from part 3.3.4.2 show a greater preference for nanoplankton (0.56 ± 0.04). The omnivorous and phytoplankton-feeding-biased Cladocerans have a greater preference for picoplankton (0.76 ± 0.05). The omnivorous and phytoplankton-feeding-biased Tunicates show preferences for both nanoplankton (0.48 ± 0.05) and picoplankton (0.47 ± 0.05). In the diet of the omnivorous *N. scintillans*, Copepodites account for the largest proportion, which is 0.64 ± 0.07. Other crustaceans prefer Copepods (0.84 ± 0.07). Planktonic larvae prefer to feed on Cladocerans (0.58 ± 0.14). Other plankton prefer to feed on Copepodites (0.68 ± 0.04).

#### 3.4.2. Food Composition and Feeding Preferences of Mesozooplankton Species

**Figure 11:**
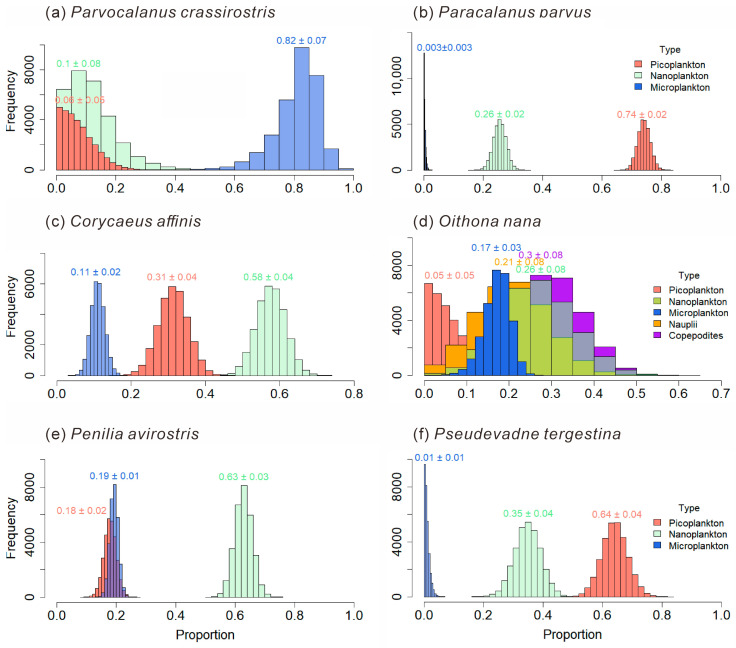
Food composition of main mesozooplankton species: *Parvocalanus crassirostris* (**a**); *Paracalanus parvus* (**b**); *Corycaeus affnis* (**c**); *Oithona nana* (**d**); *Penilia avirostris* (**e**); *Pseudevadne tergestina* (**f**).

The food composition of mesozooplankton species is shown in Figure 11. In the present analysis, bacterial biomass is operationally encompassed within the picoplankton size fraction. The carnivorous *P. crassirostris* feeds on Microplankton the most, accounting for 0.82 ± 0.07 of its diet. The phytoplankton-feeding-biased *Paracalanus parvus* selectively feeds more on picoplankton, which accounts for 0.74 ± 0.02 of the diet of *P. parvus*. The phytoplankton-feeding-biased *Corycaeus affinis* selectively feeds more on Nanoplankton (0.58 ± 0.04). The carnivorous *O. nana* has a relatively higher trophic level, and its diet is more complex. The omnivorous and phytoplankton-feeding-biased *P. avirostris* in Cladocerans has a greater preference for nanoplankton (0.63 ± 0.03). While the carnivorous *Pseudevadne tergestina* in Cladocerans prefers to feed more on picoplankton (0.64 ± 0.04).

## 4. Discussion

### 4.1. Spatiotemporal Variations and Environmental Drivers of the Mesozooplankton Community Structure in Daya Bay

The findings of this study reveal pronounced spatiotemporal variations in the mesozooplankton community structure within the study area. Spatially, the northern region, characterized by elevated temperatures associated with thermal discharge, generally exhibited lower mesozooplankton abundance and diversity indices (Figure 4). This pattern is consistent with the significant negative correlations observed between temperature and key community metrics, including total abundance and diversity indices (Figure 7), suggesting that thermal discharge may exert a suppressive influence on mesozooplankton communities.

Seasonally, substantial variations were evident in abundance, diversity indices, species composition, and dominant taxa. The most distinctive feature of the spring assemblage was the marked dominance of cladocerans and tunicates, whose relative abundance peaked among all four seasons (Figure 5a,e). The dominant species included the cladoceran *P. avirostris* and the tunicate *D. gegenbauri*, both recognized as typical warm-water species [31,32]. Previous studies have reported that thaliaceans, including *D. gegenbauri*, are oceanic zooplankton whose distribution in Daya Bay is largely governed by offshore water intrusion and primarily regulated by temperature [33]. Earlier surveys in Daya Bay indicated that thaliaceans were only frequently encountered during summer [34]. Similarly, *P. avirostris* was previously identified as a summer-dominant species in Daya Bay [35]. However, more recent observations have documented an earlier seasonal occurrence, with both species now becoming dominant in spring [32]. The present study corroborates this phenological shift and suggests that the rising surface seawater temperature in Daya Bay, driven by the combined effects of global warming and thermal discharge [36,37], may be driving seasonal changes in the composition of dominant mesozooplankton species.

The dinoflagellate *N. scintillans* dominated overwhelmingly during both summer and winter, accounting for more than 50% of total mesozooplankton abundance (Figure 5e). Peak abundances reached 203,320.42 ind.m^−3^ in summer and 36,048.59 ind.m^−3^ in winter, indicating the occurrence of bloom events. Concurrently, significantly elevated concentrations of Nitrate, Ammonia, and NanoChl *a* were recorded during these seasons, suggesting enhanced nutrient levels and elevated chlorophyll *a* concentrations that favored *N. scintillans* proliferation. In recent years, *N. scintillans* blooms have been increasingly reported in Daya Bay [38,39], and the observation of such blooms in two of the four cruises in this study further supports the ongoing intensification of eutrophication in this region [5]. Hierarchical partitioning based on redundancy analysis identified Nitrate as the most influential environmental factor shaping community differentiation (Figure 6b), underscoring the central regulatory role of nutrient enrichment.

Overall, the results demonstrate that the spatiotemporal dynamics of mesozooplankton are closely linked to anthropogenic influences, including thermal discharge, climate warming, and eutrophication, suggesting that human activities have already exerted substantial impacts on the coastal planktonic ecosystem. Continued long-term ecological monitoring will be essential to clarify how temperature and nutrient interactions drive community succession.

### 4.2. Feeding Habits and Selectivity of Dominant Mesozooplankton Groups

The diverse feeding preferences exhibited by the zooplankton taxa in this study provide important insights into their ecological functions and the structuring of pelagic food webs.

Copepods, major contributors to omnivorous mesozooplankton communities, are generally omnivorous but show a preference for nanoplankton (Figure 10a). Different taxa exhibit distinct feeding habits and dietary preferences. The order Calanoida, which dominated across all four seasons, showed an herbivorous tendency, consistent with previous studies [22]. Our study further revealed species-specific feeding selectivity of Calanoida, for example, *P. crassirostris* primarily consumed microplankton, whereas *P. parvus* preferred picoplankton (Figure 11a,b). Cyclopoida and Harpacticoida exhibited an omnivorous-to-carnivorous feeding tendency, occupying higher trophic levels than Calanoida (Figure 9a). Some species of Cyclopoida and Harpacticoida are carnivorous, such as the genera Clytemnestra and Macrosetella (Figure 9b). Their dominance in autumn (Figure 5e) suggests a shift toward higher trophic levels within the mesozooplankton community. Such trophic positioning may influence community structure and dynamics, potentially triggering trophic cascades that regulate the abundance of phytoplankton-feeding mesozooplankton and, in turn, indirectly affect phytoplankton growth and community composition [40]. These findings underscore the trophic diversity within copepod assemblages and highlight their complex ecological roles in maintaining community stability and ecosystem functioning.

This study revealed that cladocerans are generally omnivorous with a tendency toward herbivory, exhibiting a distinct preference for picoplankton, which includes bacteria (Figure 10b). In particular, *P. avirostris* showed a stronger preference for nanoplankton (Figure 11e), whereas the carnivorous *P. tergestina* tended to consume a higher proportion of picoplankton (Figure 11f). This result is consistent with previous studies [41,42]. In terms of trophic positioning, *P. avirostris* occupies a lower trophic level than *P. tergestina* (Figure 9b), a pattern that is consistent across different regions [41,42,43]. A previous study in Daya Bay further suggested that *P. avirostris* consumed a higher percentage of phytoplankton with the seasonal transition [43], supporting its relatively low trophic status observed in this study.

Tunicates, as highly efficient filter-feeding zooplankton, have traditionally been regarded as non-selective feeders [44,45]. However, recent studies have challenged this paradigm by revealing discrepancies between the proportions of prey types in tunicate diets and their availability in the surrounding environment [46,47,48]. Our results provide additional evidence for selective feeding in tunicates, revealing a marked preference for nanoplankton and picoplankton (Figure 10c). The increased abundance and dominance of herbivorous cladocerans and tunicates in spring (Figure 5e) likely enhanced top-down control on phytoplankton, leading to the reduction in nanoChl *a* and totalChl *a* observed during this period (Figure 3a).

The heterotrophic dinoflagellate *N. scintillans* was identified as an omnivorous zooplankton species, consistent with previous observations [38]. Its diet was dominated by copepod larvae (Figure 10d), and its elevated δ^13^C and δ^15^N values across seasons reflected a relatively high trophic position (Figure 8b). As a high-trophic-level omnivore, *N. scintillans* exerts dual ecological effects: it suppresses phytoplankton through direct grazing while also preying on zooplankton eggs and larvae and competing for food resources. These interactions collectively impose negative pressures on other plankton populations, particularly during bloom events [49,50]. In this study, *N. scintillans* blooms in summer and winter coincided with significant declines in zooplankton diversity indices, indicating an immediate ecological response to its proliferation. Given that copepod larvae constituted the major component of its diet, such blooms likely further constrained copepod population development, inducing pronounced trophic cascades and reshaping zooplankton community structure in Daya Bay. These findings highlight the importance of monitoring eutrophication-driven *N. scintillans* blooms and their cascading ecological impacts.

In summary, environmentally induced shifts in dominant zooplankton taxa with differing trophic strategies can exert contrasting effects on community structure and ecosystem functioning. Liu et al. [51] reported evident mesozooplankton selective feeding behavior and corresponding variations in the strength of trophic cascades on phytoplankton in Daya Bay with high cladoceran abundance led to high feeding rates while high omnivorous copepods abundance intensified high trophic cascades on small-sized phytoplankton.

### 4.3. Trophic Structure in the Thermal Discharge Area of Daya Bay Nuclear Power Plant

This study elucidates a multi-level trophic structure within the planktonic food web of the Daya Bay Nuclear Power Plant area. The food web is structured around size-fractionated phytoplankton as basal resources, omnivorous mesozooplankton as central energy conduits, and carnivorous zooplankton as upper-trophic consumers.

The trophic hierarchy begins with Picoplankton (0–2 μm) serving as the foundational energy source (TL ≈ 1). Nano- (2–20 μm) and micro-plankton (20–200 μm) occupy intermediate basal positions (TL ≈ 1.5–2.0), functioning both as primary producers and primary consumers, and channel energy to mesozooplankton. The core intermediate trophic levels (TL ≈ 2.0–2.5) are dominated by omnivorous mesozooplankton such as calanoid copepods (e.g., *Paracalanus* and *Acartia*), cladocerans (*P. avirostris*), and tunicates (*D. gegenbauri*). These groups exhibit flexible feeding strategies, consuming both autotrophic and heterotrophic prey, thereby enhancing trophic connectivity and stabilizing energy transfer [41,52,53]. Higher trophic positions (TL ≈ 2.5–3.5) are occupied by carnivorous zooplankton including most cyclopoid and harpacticoid copepods as well as a carnivorous calanoid genus (*Tortanus*) and the heterotrophic dinoflagellate *N. scintillans*. The apex of the mesozooplankton food web (TL > 3.5) includes other crustaceans (e.g., ostracods, decapods) and planktonic larvae, which prey extensively on smaller zooplankton and serve as top predators.

The results of this study indicate that most mesozooplankton groups occupy the main intermediate trophic levels and exhibit omnivorous feeding behavior. The dominance of omnivory in this food web enhances ecosystem resilience by broadening energy pathways and providing trophic redundancy. This configuration supports more stable control of phytoplankton populations and facilitates efficient nutrient recycling [14,54]. Their mixed feeding on both autotrophic and heterotrophic prey broadens energy transfer pathways and enhances trophic connectivity, which helps buffer the system against environmental fluctuations and promotes more balanced and sustained control of phytoplankton.

This trophic structure holds special significance in the thermal discharge area of Daya Bay Nuclear Power Plant. It reflects the adaptation of the plankton community in this area to the environment and the maintenance of ecological balance. Compared with other marine regions, the Daya Bay food web shares a similar hierarchical organization but exhibits distinct energetic adaptations driven by local anthropogenic pressures. Continuous thermal discharge modifies zooplankton feeding preferences and trophic relationships through temperature-mediated effects on metabolism and prey selection. For instance, elevated temperatures may shift some omnivorous feeders toward greater carnivory, thereby altering energy flow patterns and strengthening top-down control mechanisms. This is consistent with observations in Gwangyang Bay, where rising water temperatures have been linked to shifts in zooplankton community structure and trophic interactions [22]. Moreover, thermal discharge induced changes in local food conditions indirectly affect the food acquisition of zooplankton, thereby influencing their feeding selectivity and the overall food web structure [51].

Beyond the phytoplankton-based energy pathways, detritus constitutes a significant supplementary energy source, particularly during periods of low primary production. Tunicates and harpacticoid copepods generally emerge as key detritivores, efficiently converting particulate organic matter into mesozooplankton biomass [55,56]. This detrital pathway enhances food web stability by providing an alternative energy conduit when phytoplankton resources are limited, thereby buffering the system against seasonal fluctuations in primary production. The flexibility of omnivorous mesozooplankton to utilize detrital resources represents an important adaptation to the variable environmental conditions characteristic of the Daya Bay ecosystem. Although not directly measured in this study, the relatively depleted δ^13^C values observed in some groups (−25‰ to −26‰) may indicate detrital carbon incorporation. Future studies should explicitly quantify the contribution of detritus to the mesozooplankton diet in Daya Bay.

## 5. Conclusions

This study elucidates the spatiotemporal dynamics, feeding ecology, and trophic structure of mesozooplankton in Daya Bay, revealing robust linkages between environmental forcing and community organization. Spatially, thermal discharge strongly influenced the mesozooplankton community structure, suppressing mesozooplankton abundance and diversity through elevated temperatures. Seasonally, species composition shifts—such as the dominance of *P. avirostris* and *D. gegenbauri* in spring and recurrent *N. scintillans* blooms in summer and winter—underscore the combined impacts of anthropogenic stressors on phenology and community assembly.

Trophic strategies diverged markedly among dominant taxa. Copepods exhibited omnivorous habits with varying selectivity for pico- and nanoplankton, whereas cladocerans and tunicates displayed stronger herbivorous tendencies for smaller phytoplankton. In contrast, *N. scintillans* functioned as a high-trophic-level omnivore, preying on copepod nauplii while competing for shared resources. These contrasting energetic pathways shape trophic interactions and mediate community stability.

These findings highlight the adaptive capacity of the planktonic food web to anthropogenic pressures. The prevalence of omnivory and functional differentiation among zooplankton functional groups confer trophic flexibility and redundancy—critical mechanisms for maintaining ecosystem stability in changing environments. This complexity demands integrated management strategies for coastal conservation. Long-term monitoring and mechanistic experiments are essential to predict how continuing human activities will reconfigure planktonic food web dynamics in subtropical coastal ecosystems.

## Data Availability

The original contributions presented in this study are included in the article. Further inquiries can be directed to the corresponding author (Mianrun Chen).
